# A randomized controlled trial of well-being therapy to promote adaptation and alleviate emotional distress among medical freshmen

**DOI:** 10.1186/s12909-019-1616-9

**Published:** 2019-06-03

**Authors:** Yuan-Yuan Xu, Tong Wu, Yong-Ju Yu, Min Li

**Affiliations:** Department of Military Psychology, College of Psychology, Third Military Medical University, 30 Gaotanyan Main St, Chongqing, 400038 China

**Keywords:** Psychological well-being, Adaptation, Depression, Anxiety, First year medical students

## Abstract

**Background:**

Maladjustment and emotional distress are extremely prevalent among first-year medical students in college and are associated with numerous negative consequences for medical freshmen, their families and universities. The current research aimed to detect the efficacy of a well-being therapy in promoting adaptation to college life and alleviating emotional distress among medical freshmen.

**Methods:**

One hundred one participants who met the inclusion criteria were enrolled in a single-blind randomized controlled trial. Well-being therapy was given to the intervention group weekly for 5 weeks (WBT, *n* = 50). At the same time, students in the placebo control condition (CC, *n* = 51) were required to record early memory for 5 weeks and at weekly meetings it would be shared voluntarily. Psychological well-being, adaptation, anxiety and depression were recorded at pretest, posttest, and at three-month follow-up. Data from 87 first-year students with complete follow-ups (WBT, *n* = 39; CC, *n* = 48) were analyzed over three time periods.

**Results:**

Compared with the control group, students undergoing the 5-week well-being therapy reported larger improvements in psychological well-being and adaptation, and greater alleviation in symptoms of anxiety and depression from pretest to posttest to follow-up.

**Conclusions:**

Well-being intervention may provide first-year medical students with skills to efficiently manage maladjustment and emotional distress. It seems that medical freshmen would benefit a lot when such an intervention programme could be incorporated into the general medical education.

**Trial registration number:**

ChiCTR-ROC-17012636. Registered 11 September 2017 (Retrospectively registered) at Chinese Clinical Trial Registry.

## Background

It is well-documented that maladjustment and emotional distress are extremely prevalent among college freshmen [1]. In addition, the heavy tasks of medical learning further place first-year medical students at greater risk of experiencing emotional distress, such as depression and anxiety, and showing maladaptive symptoms when compared to other college students [2, 3]. According to previous research, approximately 20.9% of Chinese medical students suffer high levels of depression, and the prevalence of anxiety among this group is approximately 19.6% [4]. Numerous harmful consequences are associated with maladjustment and psychological distress among medical freshmen, and every year there are some medical freshmen risk becoming incapable of performing the tasks of daily life or even drop out of school due to these maladaptive symptoms [5].

A large body of literature suggests that the facilitation of well-being and optimal human functioning could be a desirable strategy for decreasing the intensity of psychobiological distress and might yield crucial protective factors when confronted with challenges and adversity, and this has been confirmed in randomized controlled studies [6, 7]. For example, pilot studies were previously carried out in Italy to detect the effect of a well-being enhancement programme on middle and high school students [6, 8]. It was found that depression, anxiety and somatization improved significantly after attending the programme. Due to the promising results collected from middle and high school students, the crucial intention of the present research was to examine a well-being training aimed at promoting adaptation to college life and alleviating emotional distress for medical freshmen.

Psychological adaptation refers to the processes by which individuals have active responses to the environment and further establish a balanced relationship with it [9]. For many college freshmen, especially those in China, going to university involves a string of life and academic challenges, such as a greatly transformed learning pattern (i.e., from passive learning to active learning), complicated interpersonal relationships (e.g., with roommates and tutors) and unfamiliar circumstances (e.g. diets, climate and lifestyle habits) [1]. In addition, along with the arduous tasks of medical learning, including heavy workloads, tight time schedules, and the dissection of corpses, first-year medical students are prone to suffering from increased study-related stressors [3]. Maladjustment occurs when the students fail to address these challenges, resulting in detriments in their physical and mental health [10]. Emotional distress might be conceptualized as a particular form of maladjustment [11]. Research concentrating on mental distress owing to long-term exposure to stressors revealed increased depression and anxiety in medical freshmen compared to ordinary people [3].

Emotional distress in medical students has been correlated with substance abuse, broken relationships [2], suicidal ideation and serious thoughts of dropping out [5]. At the organizational level, emotional distress has been correlated with a series of adverse problems, such as increased risk of brain drain, reduced productivity [12, 13], and higher manpower and material resources input to guarantee the safety of students. Interventions to promote adaptation to college life and studies and to alleviate emotional distress among first-year medical students are therefore important at the personal level and the organizational level.

Research has confirmed that an improvement of psychological well-being (PWB) can contribute to enhancing individuals’ coping capacity and resilience in the face of stress, thus preventing negative emotions from spiralling downwards into clinical disorders [14]. From this perspective, PWB may hold considerable promise for promoting adaptation and reducing emotional distress among medical freshmen. According to Ryff and her colleagues [7], PWB involves processes of experiencing deep connections with others, trying to identify one’s potential, setting and pursuing goals, regulating demands and opportunities around, and experiencing self-acceptance and self-determination. All of these properties are supposed to benefit maladaptive medical freshmen. The effect of PWB in improving emotional distress in general [15, 16] and on the symptoms of various mental disorders [17–19] has been well demonstrated. In addition, PWB has also been effective in cultivating approach-oriented coping strategies among individuals experiencing high stress [20]. As such, psychological well-being intervention may be an efficient solution for improving maladjustment and emotional distress among medical freshmen.

One promising approach to boosting psychological well-being is through Fava’s “well-being therapy” (WBT) [21, 22], which is based on Ryff’s multidimensional model of PWB, encompassing 6 dimensions: autonomy, personal growth, environmental mastery, purpose in life, positive relations and self-acceptance [7, 21]. Previous study has shown the long-term benefits of WBT in preventing recurrent depression [23] and anxiety [24] in clinical populations. Subsequent research extended the approach to preventing emotional distress in school condition and boosting optimal human functioning among teenagers [6]. However, such intervention has not investigated medical freshmen, who have high levels of distress.

The current research aimed to investigate the efficacy of WBT to promote adaptation and alleviate emotional distress among first-year medical students. This population was selected because maladjustment and emotional distress are especially prevalent among them. It was hypothesized that students in a WBT condition would report greater improvement in adaptation and less emotional distress at the end of the intervention and 3months later, compared with students in the placebo control condition.

## Methods

### Design

This was a two-arm (a WBT condition and a placebo control condition), randomized controlled trial including 3 test periods (pre-test, post-test and 3-month follow-up). The design was to analyze the training effects in comparison to placebo controls both in the short term and long term.

This study was carried out in accordance with the Declaration of Helsinki and was approved by the Ethics Committee of the Third Military Medical University with written informed consent from all subjects. The protocol adheres to CONSORT guidelines.

### Participants and recruitment

Recruitment advertisements were posted around the freshman dorms in a medical university to invite all students in first year (*n* = 550). Students who signed up for the intervention were required to complete the screening questionnaires (*n* = 238), and were enrolled under the following criteria: ① being freshmen of medical major,② with no experience of psychological intervention, ③ participating in no other psychological intervention in the training period. The research objectives and design were then offered to the qualified students, with written informed consent obtained from all of them. After the preassessment and screening questionnaires, students who satisfied all the condition (*n* = 101) were assinged to the WBT (*n* = 50) or the CC (*n* = 51) randomly. The attrition rates of the intervention and control group were 22% (8 dropped out because of sporadic attendance, 2 discontinued intervention due to their training in campus broadcasting, and 1 got a left knee injury) and 5.88% (3 dropped out because of Student Union business), respectively.

### Measures

The training was evaluated in terms of changes correlated with students’ psychological well-being, adaptation and traditional negative indicators of mental distress (such as depression and anxiety).

#### Well-being

Well-being was measured using the Chinese version of Ryff’s scales of psychological well-being (RPWB) [25]. The RPWB consists of 18 items, which assesses autonomy, environmental mastery, personal growth, positive relations with others, purpose in life, and self-acceptance. Each item is rated on a six-point Likert scale from 1 (strongly disagree) to 6 (strongly agree) to rate the degree to which they agreed or disagreed with, with higher scores indicating greater psychological well-being. In the current study, the reliability coefficient alpha was 0.969 for total scale, and Cronbach’s alpha estimates of the six dimensions ranged from 0.858 to 0.959.

#### Adaptation

The validated Chinese college student adjustment scale (CCSAS) [26] was used to measure the level of adaptation. The CCSAS is a 60-item questionnaire assessing adaptation along seven dimensions: interpersonal relationship adaption, learning adaption, campus life adaption, job-seeking adaption, emotional adaption, self-adaption and satisfaction. Students were invited to rate the degree to which they agreed or disagreed with each item on a five-point Likert scale, from 1 (disagree) to 5 (agree), with higher scores indicating better adaptability. In the current sample, the reliability coefficient was 0.955 for total scale, and Cronbach’s alpha estimates of the seven dimensions ranged from 0.694 to 0.848.

#### Emotional distress

The validated Chinese version of the centre for epidemiological studies depression scale (CES-D) [27] was used to measure the level of depression. It is a self-report questionnaire with 20 items that is used widely to measure the current level of depressive symptomatology in the general population. Each item is rated on a four-point Likert scale, from 0 (occasionally or never) to 3 (most of the time or continuously) to evaluate the frequency of occurrence of the symptoms. Total scores ranged from 0 to 60, with higher scores indicating more depression. The Cronbach’s alpha coefficient for our sample was 0.905.

Anxiety was measured using the 20-item validated Chinese version of the state anxiety inventory (SAI) [28] to the level of anxiety. Students were invited to evaluate some certain subjective feelings during the recent week on a four-point Likert scale, from “not at all” (1) to “very much so” (4). Subjective feelings contain worries, nervousness and fear for the time being or in a certain period of time are thus measured. The Cronbach’s alpha coefficient was 0.935.

### Procedure

**Fig. 1:**
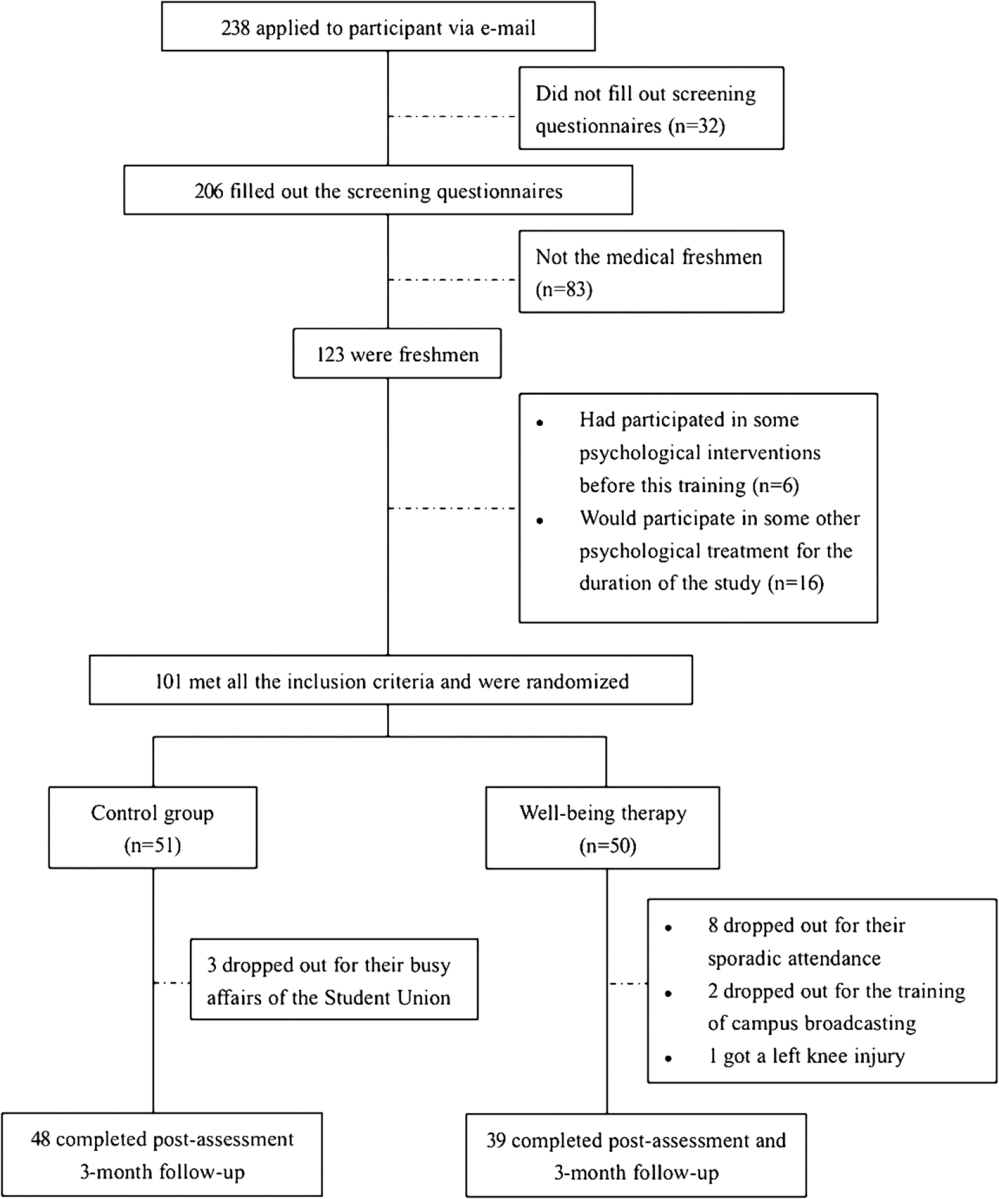
Flow of participants

In order to fit within the school term and training objectives, an elective course named “Psychology and Life” was set to conduct the intervention. Students in the intervention group (three classes, 16-17 students per class) received WBT once a week for 5 weeks. Participants in the control condition (three classes, 17 students per class) were invited to take note of the early-day memories for 5 weeks, and to share once a week in class. Both five-session trainings were conducted by 3 professional psychologists. Although participants were blinded to the group assignment, the practitioners are well-informed to know the experimental procedure since the entire process requires skillful manipulation, thus the study was a randomized single-blinded control trial. Outcome measures were gathered over the Internet immediately after the training and 3 months later (see Fig. 1).

### Intervention

#### Well-being therapy

The five-session, 2-h-per-session intervention was an integration of psychological well-being education and practice. Each session contained group discussions of the previous week’s exercises (except session 1), introductions to the current session’s content and concepts relevant to well-being, and homework assignments that involved recording the circumstances surrounding some topics in a structured diary and implementing the learned skills in daily life. The first session contained an introduction to the Ryff’s multidimensional model of psychological well-being. In the second session, participants were required to write down the events of well-being in a structured diary, in terms of environmental mastery, personal growth, purpose in life, autonomy, self-acceptance and positive relations with others [7, 21] (e.g., I had a nice weekend with roommates and they brought me a lot of happiness). When the students are capable of recognizing the incidents of well-being, they are further inspired to discern the thoughts and beliefs leading to the premature interpretation of well-being [21] (e.g., just because I paid for the dinner), and challenge these thoughts with appropriate refutations (e.g., they like me) in the third session. The supervising of the process of well-being episodes makes it possible for the professionals to certify particular impairments in well-being factors in accordance with Ryff’s conceptual structure, and then to discuss them with students in the fourth session. In addition, participants are also encouraged to reinforce positive exercises that are possible to boost well-being for a certain time each day [21] in this session. Reflection on the implementation of these skills in real life was explored in the fifth session, with a review of the practice and theoretical framework in mental health improvement (see Table 1).

**Table 1:** Session by session description of interventions

Session	Well-being therapy	Placebo control condition
1	Get to know each other. Introduce the Ryff’s multidimensional model of psychological well-being. Homework: record the circumstances surrounding the episodes of well-being in a structured diary, encompassing environmental mastery, personal growth, purpose in life, autonomy, self-acceptance and positive relations with others (e.g. I had a nice weekend with roommates and they brought me a lot of happiness).	Get to know each other. Students are encouraged to recall and share their early memories about environmental mastery. One occasion that students would become “much happier and satisfied with their lives” is introduced. Homework: write down something from their early memories about personal growth in a structured diary.
2	Students are encouraged to share the records of well-being episodes and their feeling about these things (e.g. Which kind of well-being components brought the greatest happiness experience?). Homework: record their interpretation of the well-being events (e.g. Just because I paid for the dinner).	Students are encouraged to share their records about personal growth. One occasion that students would become “much happier and satisfied with their lives” is introduced. Homework: write down something from their early memories about life purpose in a structured diary.
3	Students are encouraged to identify thoughts and beliefs leading to premature interpretation of well-being and challenge these thoughts with appropriate refute (e.g. They like me). Homework: implement the learned optimal thoughts and beliefs in daily life.	Students are encouraged to share their records about life purpose. One occasion that students would become “much happier and satisfied with their lives” is introduced. Homework: write down something from their early memories about autonomy and self-appraisal in a structured diary.
4	Each student’s particular impairments in well-being dimensions according to Ryff’s conceptual framework are discussed. Homework: reinforce positive activities that are likely to elicit well-being for a certain time each day	Students are encouraged to share their records about autonomy and self-appraisal. One occasion that students would become “much happier and satisfied with their lives” is introduced. Homework: write down something from their early memories about relations with others.
5	The concept of psychological well-being that integrates environmental mastery, personal growth, purpose in life, autonomy, self-acceptance and positive relations with others is discussed. Progress is reviewed, and gains and maintenance are discussed. Some advice from a therapist is given.	Students are encouraged to share their records about relations with others. One occasion that students would become “much happier and satisfied with their lives” is introduced. Progress is reviewed.

#### The placebo control condition

As for the control group, early memories regarding environmental mastery, personal growth, life purpose, autonomy, self-appraisal, or relations with others were recorded for 5 weeks and were asked to share in every class voluntarily. In addition, participants were instructed on several occasions that they would become “much happier and satisfied with their lives” at every meeting. This was done to control the possibility that any success demonstrated by the experimental group would not be an artefact of suggestion (see Table 1).

### Statistical analyses

The differences in demographic variables between the WBT group and CC group were assessed by independent samples *t* tests and Chi-square tests. The baselines of the RPWB, CCSAS, CES-D and SAI were compared using *t*-test. After splitting data by group, repeated measures analysis of variance (rANOVA) was applied to examine the discrepancies between pretest to posttest, posttest to follow-up and pretest to follow-up for the WBT group and CC group. Differences in all outcome measures between groups were analyzed using Factorial rANOVA. Bonferroni correction was applied for the rANOVA post hoc test. All analyses were performed using SPSS19.0 software, and *p*-values and partial *η*-square values (*η*_*p*_^2^) [29] were calculated for evaluating statistical significance and effect size.

The analysis was to assess the short-term and long-term effects of the training on participants’ psychological well-being, adaptation, depression and anxiety in the two conditions.

## Results

### Descriptive statistics

Among the total 550 first-year students, 238 sighed up for the intervention, contributing to a 43.27% response rate. Of those participants, 101 became eligible to participate in the study and were assigned to the intervention condition or placebo control condition. Only 14 students were not involved in follow-ups, leading to a 13.86% drop-out rate (see flow chart in Fig. 1). Table 2 shows the demographic variables of the students, and no significant difference was found between the two groups.

**Table 2:** Comparisons between the groups regarding demographic data

	Intervention group *n* = 39		Control group *n* = 48		Total *n* = 87	
	Mean	SD	Mean	SD	Mean	SD
Age	19.49	1.65	20.16	2.37	19.79	2.02
	*n*	%	*n*	%	*n*	%
Gender						
Male	21	53.85	25	52.08	46	52.87
Female	18	46.15	23	47.92	41	47.13
Family structure						
Single parent	5	12.82	8	16.67	13	14.94
Two parent	28	71.80	34	70.83	62	71.27
Other conditions	6	15.38	6	12.50	12	13.79
Origin from city or country						
City	14	35.90	19	39.58	33	37.93
Country	25	64.10	29	60.42	54	62.07

*Note*. Using Independent samples *t* tests and Chi-square tests, there was no significant difference in the demographic variables between the two groups

### Change in outcomes

Means and standard deviations for all variables for WBT and CC are showed in Table 3. The results of *t*-tests indicated that there was no significant difference on the RPWB, CCSAS, CES-D and SAI between the two study groups at pretest.

**Table 3:** Means (*M*), Standard Deviations (*SD*) for psychological well-being, adaptation, depression and anxiety at T1, T2, T3 for the WBT and CC, and the results of the repeated measures MANOVA after data were split by group

		T1 *M (SD)*	T2 *M (SD)*	T3 *M (SD)*
RPWB	WBT	88.92 (6.42)^ab^	95.85 (5.69)	95.59 (6.46)
	CC	83.99 (9.68)	90.02 (12.56)	88.75 (12.40)
CCSAS	WBT	223.87 (22.58)^ac^	249.59 (34.17)	231.23 (29.97)
	CC	226.75 (24.12)	233.54 (32.99)	227.31 (29.41)
CES-D	WBT	6.87 (6.84)	7.18 (4.45)	5.38 (4.25)
	CC	7.81 (8.22)^ab^	11.92 (9.67)	11.83 (9.96)
SAI	WBT	30.92 (9.13)	28.46 (6.00)	28.74 (6.36)
	CC	31.42 (8.25)	29.69 (6.90)	32.19 (8.34)

*Note*. *WBT* well-being therapy, *CC* control group, *T1* pre-intervention, *T2* post-intervention, *T3* Three months after the intervention, *RPWS* Chinese Version of Ryff’s Scales of Psychological Well-Being, *CCSAS* Chinese College Student Adjustment Scale, *CES-D* Centre for Epidemiological Studies Depression Scale, *SAI* State Anxiety Inventory

^a^*p*-value < 0.05 for repeated measures MANOVA post hoc test for T1-T2

^b^*p*-value < 0.05 for repeated measures MANOVA post hoc test for T1-T3

^c^*p*-value < 0.05 for repeated measures MANOVA post hoc test for T2-T3

Table 3 also demonstrated the results of rANOVA after the segmentation of data for WBT and CC across time (pretest to posttest, posttest to follow-up and pretest to follow-up). The mean values over time for each condition indicated that the RPWS and CCSAS scores of participants in the WBT significantly improved from T1 to T2, while only subtle positive effect were found in CC, during the same period. Although there were downward trends from T2 to T3 on adaptation for intervention and control groups, changes in CCSAS from T1 to T3 for both conditions were not statistically significant. With respect of emotional distress, significant increases in depression were found in the control group from T1 to T2, but no statistically significant changes from T2 to T3 were observed. There was no significant difference in overall change for the intervention condition from T1 to T2 to T3 on depression. Changes in SAI from T1 to T2 to T3 for both groups were not statistically significant.

**Fig. 2:**
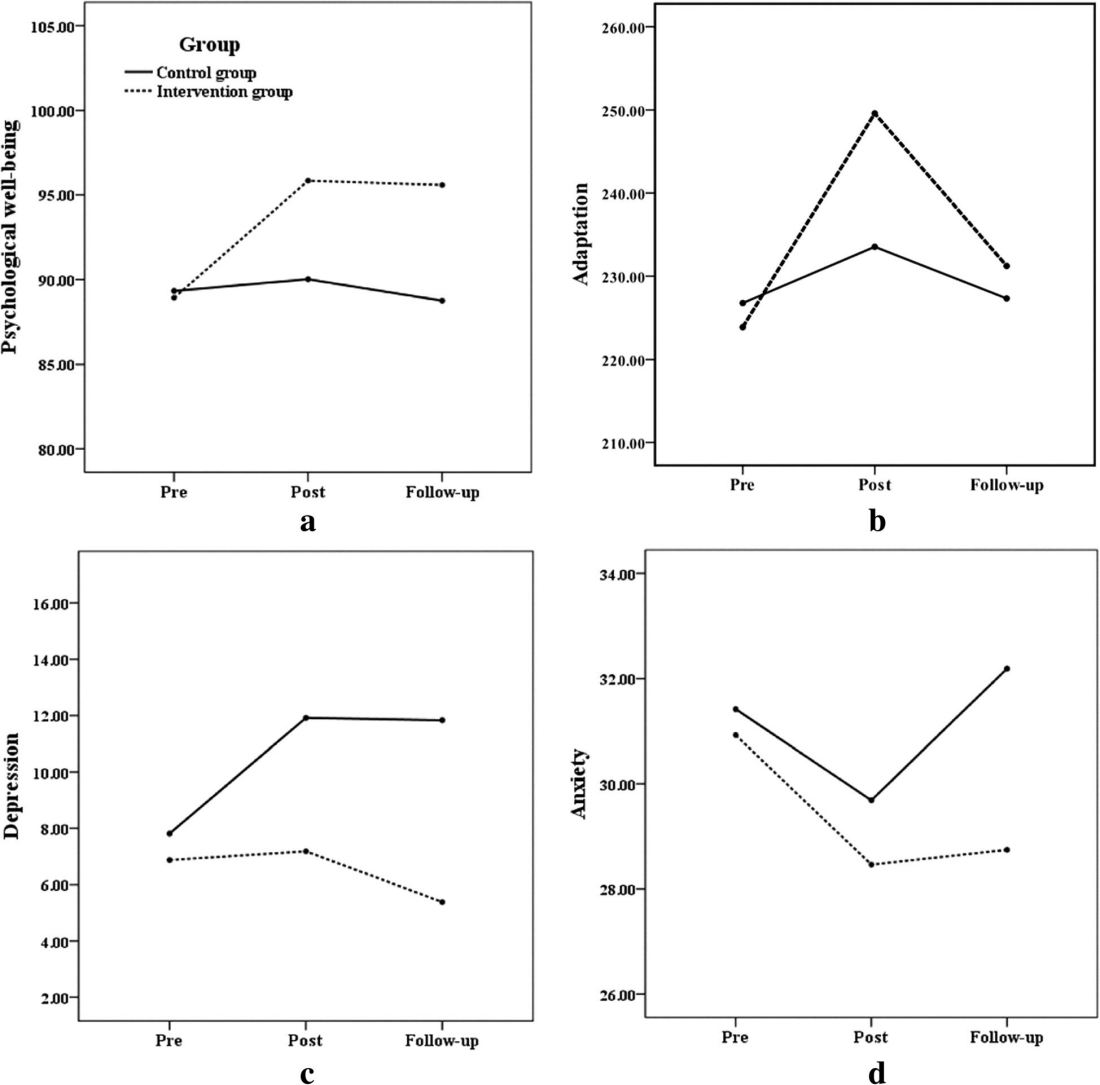
Participants’ mean levels of outcome measures (**a**. Psychological well-being; **b**. Adaptation; **c**. Depression; **d**. Anxiety) at baseline, post-intervention, and three-month follow-up

Factorial rANOVA results and the post hoc tests are detailed in Table 4. Psychological well-being, adaptation, depression and anxiety measures across time are presented in Fig. 2a, b, c and d, respectively. According to Table 4, the interaction between time and group were only significant in terms of RPWB, CCSAS, and CES-D. More specifically, combined with the results in Table 3, training effects at posttest and 3- month follow-up for RPWB and CES-D were significant, with higher levels of psychological well-being and lower levels of depression in the intervention group (see Fig. 2a and c, respectively). In addition, the significant training effects were also observed at posttest for CCSAS, with a higher level of adaptation in the intervention group (see Fig. 2b). Results of a Factorial rANOVA analysis on the anxiety measure of the matched samples from T1 to T2 to T3 showed that the interaction between time and group were not significant. Although patterns of overall change did not statistically differ between groups, there were statistically significant between-group differences at T3, with lower levels of anxiety in the intervention group (see Fig. 2d).

**Table 4:** Factorial rANOVA and post hoc test results for the interaction of groups and psychological well-being, adaptation, depression and anxiety

	Within subject effect *F*, *p*,*η*_*p*_^*2*^	Between subject effect *F*, *p*,*η*_*p*_^*2*^	Post hoc test time × group *F*, *p*,*η*_*p*_^*2*^
RPWB	*F* = 7.13, *p* < 0.01,*η*_*p*_^*2*^ = 0.08	*F* = 6.06, *p* = 0.02,*η*_*p*_^*2*^ = 0.08	
T1-T2 × group			*F* = 9.58, *p* < 0.01,*η*_*p*_^*2*^ = 0.10
T2-T3 × group			*F* = 0.22, *p* = 0.64,*η*_*p*_^*2*^ < 0.01
T1-T3 × group			*F* = 10.58, *p* < 0.01,*η*_*p*_^*2*^ = 0.11
CCSAS	*F* = 9.88, *p* < 0.01,*η*_*p*_^*2*^ = 0.10	*F* = 1.61, *p* = 0.21,*η*_*p*_^*2*^ = 0.02	
T1-T2 × group			*F* = 5.89, *p* = 0.02,*η*_*p*_^*2*^ = 0.07
T2-T3 × group			*F* = 2.16, *p* = 0.15,*η*_*p*_^*2*^ = 0.03
T1-T3 × group			*F* = 1.01, *p* = 0.32,*η*_*p*_^*2*^ = 0.01
CES-D	*F* = 4.05, *p* = 0.02,*η*_*p*_^*2*^ = 0.05	*F* = 8.13, *p* = 0.01,*η*_*p*_^*2*^ = 0.09	
T1-T2 × group			*F* = 6.17, *p* = 0.02,*η*_*p*_^*2*^ = 0.07
T2-T3 × group			*F* = 1.28, *p* = 0.26,*η*_*p*_^*2*^ = 0.02
T1-T3 × group			*F* = 11.56, *p* < 0.01,*η*_*p*_^*2*^ = 0.12
SAI	*F* = 2.65, *p* = 0.08,*η*_*p*_^*2*^ = 0.03	*F* = 1.92, *p* = 0.17,*η*_*p*_^*2*^ = 0.02	
T1-T2 × group			*F* = 1.12, *p* = 0.66,*η*_*p*_^*2*^ < 0.01
T2-T3 × group			*F* = 1.65, *p* = 0.20,*η*_*p*_^*2*^ = 0.02
T1-T3 × group			*F* = 1.87, *p* = 0.18,*η*_*p*_^*2*^ = 0.02

*Note.T1* pre-intervention, *T2* post-intervention, *T3* Three months after the intervention, *RPWS* Chinese Version of Ryff’s Scales of Psychological Well-Being, *CCSAS* Chinese College Student Adjustment Scale, *CES-D* Centre for Epidemiological Studies Depression Scale, *SAI* State Anxiety Inventory, × refers to statistical interaction

## Discussion

This study examined the effectiveness of the well-being therapy, in comparison with a placebo control, upon the adaptation and mental health of medical freshmen in China. The results indicated that the training had a significant long-term (3 months) effect on psychological well-being and depression, and a significant short-term (immediately after the intervention) impact on adaptation on the medical freshmen compared with the CC.

### The short- and long-term effect of WBT

First, a greater improvement in psychological well-being and adaptation were found in students who underwent the 5-week well-being therapy in comparison with the control group. Over the 4 months, psychological well-being and adaptation increased immediately at posttest and 3-month follow up (though the improvement in adaptation at follow-up didn’t reach conventional levels of significance) in the training group, while only slightly positive changes were founded in the placebo control group. The results align with the strong associations between well-being and coping ability and resilience [14]. In addition, according to Ryff et al., psychological well-being could play a key protective role in preventing individuals from chronic and acute life stresses [30]. Therefore, promoting psychological well-being through school interventions could be supposed to have important positive effects on improving students’ resilience, coping style and developmental process [6].

Second, in comparison with control group, students in the intervention group reported a greater relief in depression from pretest to posttest, and this was maintained from pretest to 3-month follow up. The finding is consistent with our projection. Although changes in SAI from T1 to T2 to T3 for both groups were not statistically significant, there were statistically significant between-group differences at T3, with lower levels of anxiety in the intervention group. In general, there is a tendency for students in the control condition to report increased depression and anxiety (not significantly) during the intervention, while those of the training group experienced lessened depression and anxiety over time. The observed effects are noteworthy, in consideration of the fact that medical students are more vulnerable to the distresses in the process of freshmen adaptation to early college life, and then in the middle of the school year, when a series of tests take place and more assignments are due [31]. The results support the standpoint that such trainings could be helpful if implemented towards the early period college life or the middle of the academic term, when students’ mental health is more possibly to deteriorate [32].

### Our results compared to the literature

Since its development, the purpose of WBT was the integration of positive functioning with clinical patients’ distress [6]. Recently, it has been further used as a preventive approach for young, non-clinical individuals. Currently schools are considered the ideal condition not only for the development of learning and education, but also for the promotion of resilience and psychological well-being [6]. WBT in school settings has been extended to numerous students, especially middle and high school students, who are conceived as a more “at-risk population” for emotional disorders [33]. Tomba et al. examined the impact of a WBT on middle school students in Italy and reported a statistically significant enhancement in students’ autonomy and friendliness after the intervention [34]. Another study further investigated the long-term effect of a school-based WBT in high school students [8]. 227 participants were randomly allocated to either a well-being group or an attention control group. The results demonstrated that the well-being intervention was confirmed to be effective in boosting psychological well-being and decreasing emotional distress, and these beneficial effects of WBT were sustained for the next 6 months. This is consistent with our findings concerning with the effect on psychological well-being and adaptation, as well as the maintenance of decreases in depression or anxiety in the training group. The results are in line with previous data proving significant associations between well-being and symptom indexes. Based on the above mentioned research, we can speculated that the promotion of well-being may elicit a decrease in distress and vice versa [8].

Overall analysis results suggested that the promotion of adaptation and improvements in emotional distress (specifically depression and anxiety) can be achieved for first-year medical students and well-being therapy is one strategy to achieve this. This is remarkable in light of the apparent general benefits of well-being therapy in mentally ill populations [7, 22, 23], and there are reasons to believe that such a study can be useful for students in other years, as emotional distress is found among all medical students in all years [35]. Given that they are typically brief and accessible, proceeding by means of personal practices, group communication and discussion, WBT interventions could be implemented in medical university courses as a means of bringing long-term positive effects, both in terms of developmental processes and of prevention of distress [6]. In addition, these traits of well-being therapy may also make it a favorable choice for treating high functioning, non-disordered populations.

The present study has some limitations. Firstly, the sample size is not large enough, which possibly rejected the signification of several interactive and simple effects. Therefore, the training effects can be further verified by using a larger sample in future research. Secondly, the dropout rate of follow-up in the training condition and placebo control condition was 22 and 5.88%, respectively, which might possibly be caused by the thoughtless inclusion criteria, which failed to exclude the students with some other affairs during the same period of intervention. In upcoming studies, much more judicious inclusion criteria are recommended. In addition, To improve overall response rate and maintain students’ motivation in study, future research can resort to more approaches to recruit more students, such as monetary or credit rewards. In addition, this study limits in its single-blind nature. Large-scale population studies blind to both participants and practitioners and long-term follow-up must be taken into consideration. Lastly, the present research explored the effectiveness of the trainings only on self-reported psychological well-being; future studies should contain some other evaluation methods, such as peer-reports, objective outcomes, interviews, and so on.

## Conclusions

Above all, the current study certified the potential of well-being intervention to improve psychological well-being, promote adaptation and alleviate mental distresses. Since every year there are some medical freshmen who might become incapable of performing their daily tasks or even drop out of school from the unbearable psychological pressure or mental distress, this study has practical significance for medical universities in promoting medical students’ mental health and avoiding unwanted brain drain.

## Data Availability

The datasets generated and/or analyzed during the current study are available in the Baidu Netdisk repository, https://pan.baidu.com/s/1qYZEsSc

## Abbreviations

CCSAS: Chinese College Student Adjustment Scale
CES-D: The Centre for Epidemiological Studies Depression Scale
MANOVA: Multivariate Analysis of Variance
PWB: Psychological Well-being
RPWB: Chinese version of Ryff’s Scales of Psychological Well-Being
SAI: State Anxiety Inventory
WBT: Well-being Therapy
